# Supplementary material for: Identification of photoperiod-induced specific miRNAs in the adrenal glands of Sunite sheep (Ovis aries)
Source: Front Vet Sci. 2022 Jul 22;9:888207. doi: 10.3389/fvets.2022.888207 (PMC9354845; doi:10.3389/fvets.2022.888207)
Supplement: Supplementary file 1 [file Data_Sheet_1.ZIP › Supplementary file/miRDeep2 file.zip!/miRDeep2 file/Novel_Structure/SPLP42A3/Novel_225.pdf]

Provisional ID	:	NC_019484.2_38756
Score total	:	2.5
Score for star read(s)	:	-1.3
Score for read counts	:	0
Score for mfe	:	2.2
Score for randfold	:	1.6
Score for cons. seed	:	
Total read count	:	272
Mature read count	:	271
Loop read count	:	0
Star read count	:	1

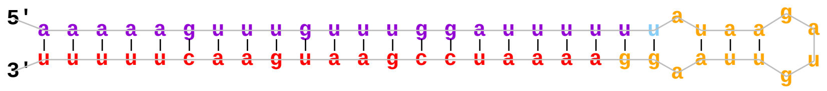

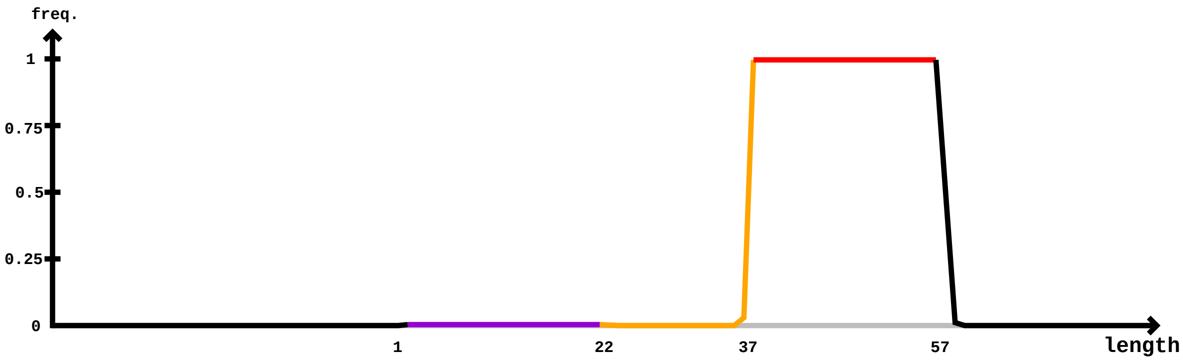


```
                                    Star                                Mature
5'- uacaugugcgaauuguuacuaauauugggcuggccaaaaaguuuguuuggauuuuuuauaagauguuaaggaaaauccgaaugaacuuuuuggccugcccaauacaucugcca  -3'  obs
    uacaugugcgaauuguuacuaauauugggcuggccaaaaaguuuguuuggauuuuuuauaagauguuaaggaaaauccgaaugaacuuuuuggccugcccaauacaucugcca       exp
    ......................((((((((.((((((((((((((((((((((((((.(((....))).)))))))))))))))))))))))))).)))))))).........   reads   mm   sample
    ...................................aaaaaguuuguuuggGuuuuu.........................................................   1       1    seq
    ......................................................................gaaaaCccgaaugaacuuuuug.....................   6       1    seq
    ......................................................................gaaaaCccgaaugaacuuuuugg....................   1       1    seq
    ......................................................................gaaaauccgaaugaacuuuuugg....................   1       0    seq
    .......................................................................aaaaucUgaaugaacuuuuu......................   247     1    seq
    .......................................................................aaaauccgaaugaacuuuuC......................   1       1    seq
    .......................................................................aaaauccgaaugaacuuuuu......................   14      0    seq
    .......................................................................aaaauccgaaugaacuuuuugg....................   1       0    seq
```
